# Solid Pseudopapillary Epithelial Neoplasm of the Pancreas: Two Cases and a Review of the Literature

**DOI:** 10.7759/cureus.116455

**Published:** 2026-09-17

**Authors:** Vishwaprasad Policepatil, Ankith Reddy Narra, Akhil Sanjay, Kishore Abuji, Vimlendra K Chaudhary, Venu Bhargava M, Manjesh KA

**Affiliations:** 1 General Surgery, Employees' State Insurance Corporation (ESIC) Medical College, Hyderabad, IND; 2 Vascular Surgery, Sree Chitra Tirunal Institute for Medical Sciences and Technology, Trivandrum, IND; 3 General Surgery, Maulana Azad Medical College, New Delhi, IND; 4 Surgical Gastroenterology, Employees' State Insurance Corporation (ESIC) Medical COllege, Hyderbad, IND

**Keywords:** mcn, non-functional pancreatic neoplasm, pancreatic neoplasm, scn, solid pseudopapillary epithelial neoplasm, spen

## Abstract

Solid pseudopapillary epithelial neoplasm (SPEN) of the pancreas is a rare cystic tumor of exocrine origin that predominantly affects young women. It may manifest with nonspecific abdominal symptoms or be discovered incidentally during imaging for unrelated conditions. We present two cases of SPEN involving the pancreatic body in young female patients, both successfully treated with curative surgical resection. Histopathological and immunohistochemical analyses in both cases confirmed the diagnosis, demonstrating characteristic pseudopapillary architecture and nuclear β-catenin positivity. Complete excision with negative margins remains the cornerstone of management, offering an excellent prognosis and long-term disease-free survival following R0 resection.

## Introduction

Solid pseudopapillary epithelial neoplasm (SPEN) is a rare pancreatic tumour accounting for approximately 1-2% of all exocrine pancreatic neoplasms [1,2]. It mainly affects young women in their second to fourth decades of life, with a notable female-to-male ratio of nearly 9:1 [3]. Although usually slow-growing and indolent, SPEN has malignant potential, with local invasion or distant metastasis reported in about 10-15% of cases [4]. The tumour’s development is closely linked to mutations in the Catenin Beta 1 (CTNNB1) gene on chromosome 3p, causing abnormal nuclear accumulation of β-catenin and loss of membranous E-cadherin expression. This activates the Wingless-related integration site (Wnt)/β-catenin signalling pathway and promotes tumour formation [5,6]. Complete surgical removal remains the preferred treatment and is often curative, with long-term survival rates exceeding 95% after total resection. The present report describes two cases of SPEN involving the pancreatic body, both of which were successfully managed through curative surgical resection. It also discusses their clinical, radiological, histopathological, and immunohistochemical features in relation to the current literature.

## Case presentation

Case one

A 26-year-old woman presented with intermittent epigastric pain and reduced appetite for eight days. Laboratory investigations, including serum amylase, lipase, and carbohydrate antigen (CA) 19.9, were within normal limits except for mild leucocytosis (Table 1).

**Table 1:** Preoperative blood investigations for the two cases Reference ranges may vary slightly depending on laboratory standards. CA: Carbohydrate antigen 19.9.

Investigation	Case one value	Case two value	Reference range
Hemoglobin (g/dL)	12.2	11.9	12–16 (Female)
Total Leukocyte Count (/µL)	24,900	16,761	4,000–11,000
Neutrophils (%)	88%	76%	40–70%
Serum Amylase (U/L)	50	1,214	30–110
Serum Lipase (U/L)	46	214	13–60
CA 19-9 (U/mL)	1.91	5	<37

Contrast-enhanced CT revealed a 5.8 cm well-defined heterogeneous solid-cystic lesion in the pancreatic body with focal calcifications, suggestive of SPEN (Figure 1).

**Figure 1:**
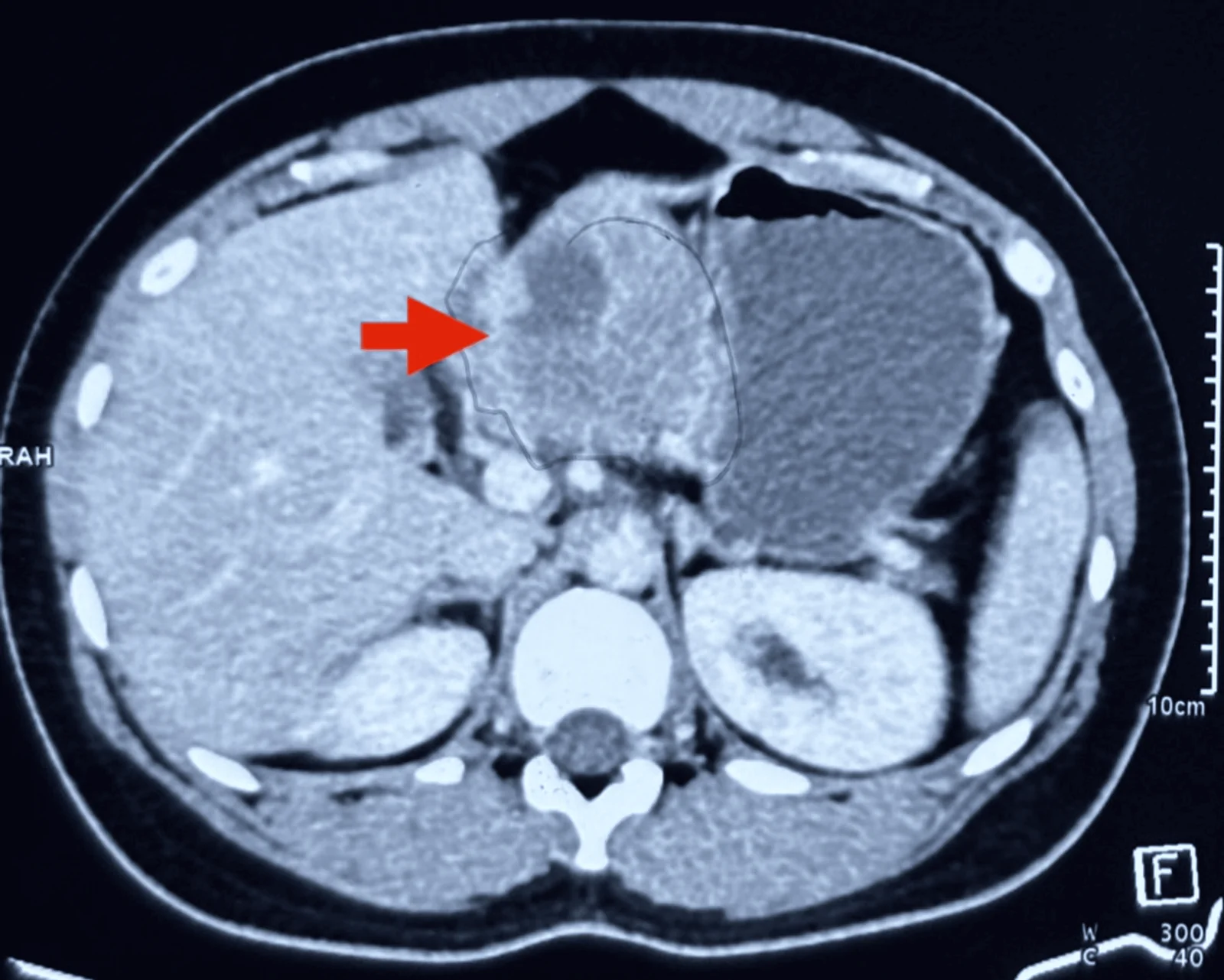
Cross sectional imaging showing large solid cystic lesion in pancreatic body with focal calcifications (red arrow indicates the tumour)

The patient underwent laparoscopic-assisted central pancreatectomy with spleen preservation (Figure 2).

**Figure 2:**
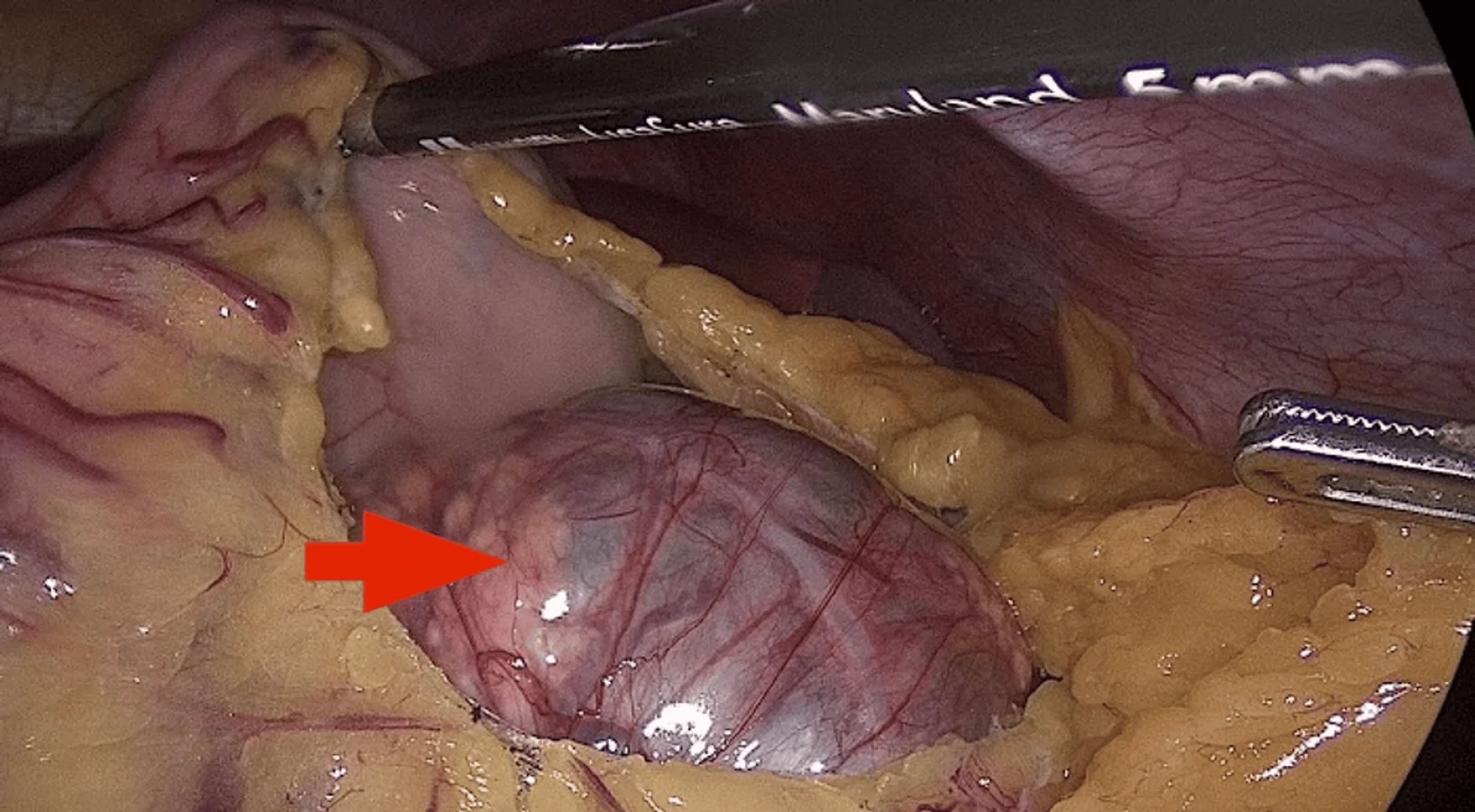
Intraoperative image showing pancreatic body lesion, solid cystic appearance (red arrow indicates the tumour)

Intraoperatively, a well-encapsulated 5 × 6 × 5 cm mass was completely excised with negative margins. Histopathology confirmed SPEN, showing pseudopapillary architecture and a characteristic immunoprofile with nuclear β-catenin positivity (Figure 3).

**Figure 3:**
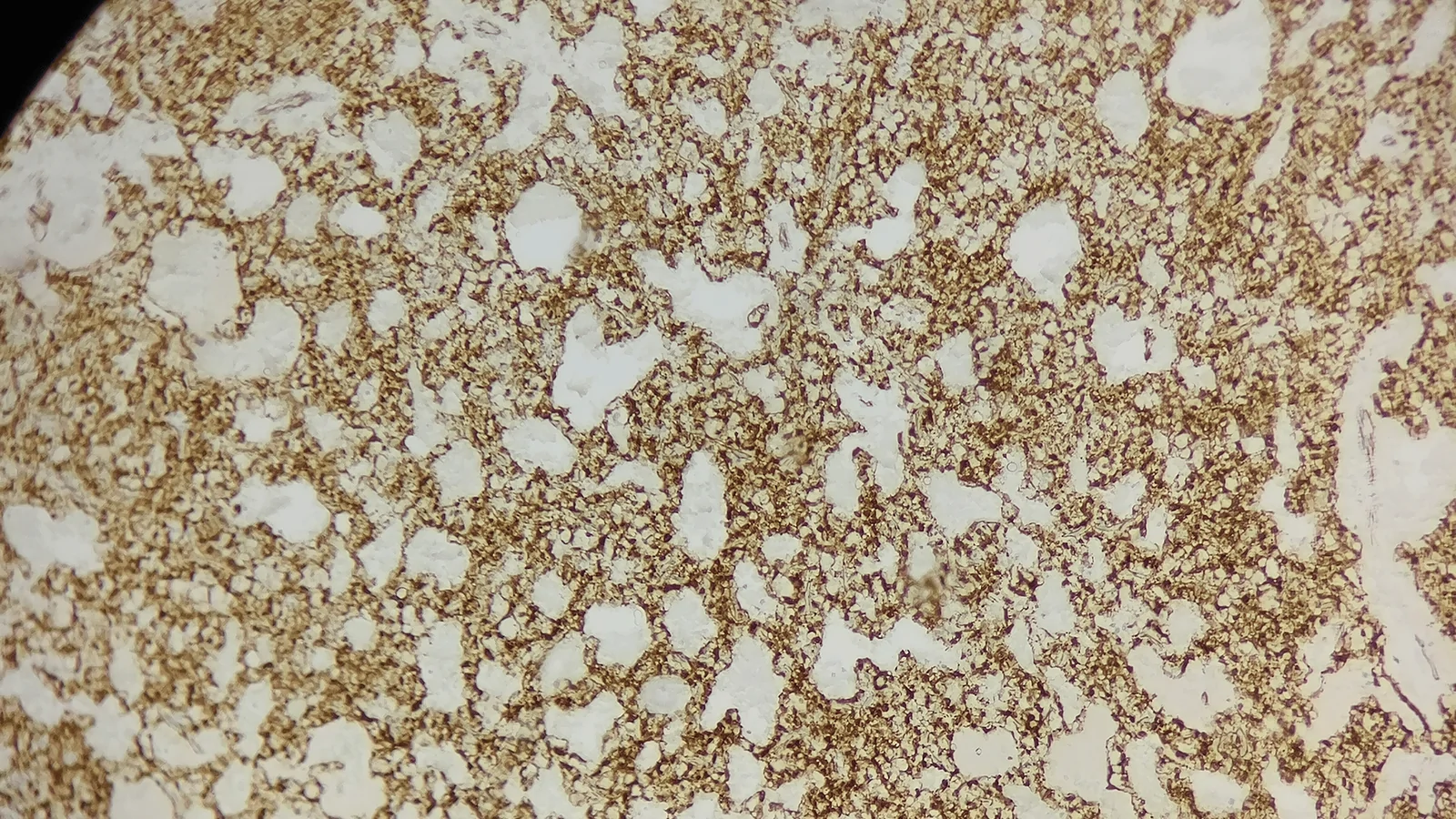
Histopatholgy image showing tumour with Beta-catenin positivity

This was along with loss of E-cadherin (Figure 4), CD10 positivity, and chromogranin negativity.

**Figure 4:**
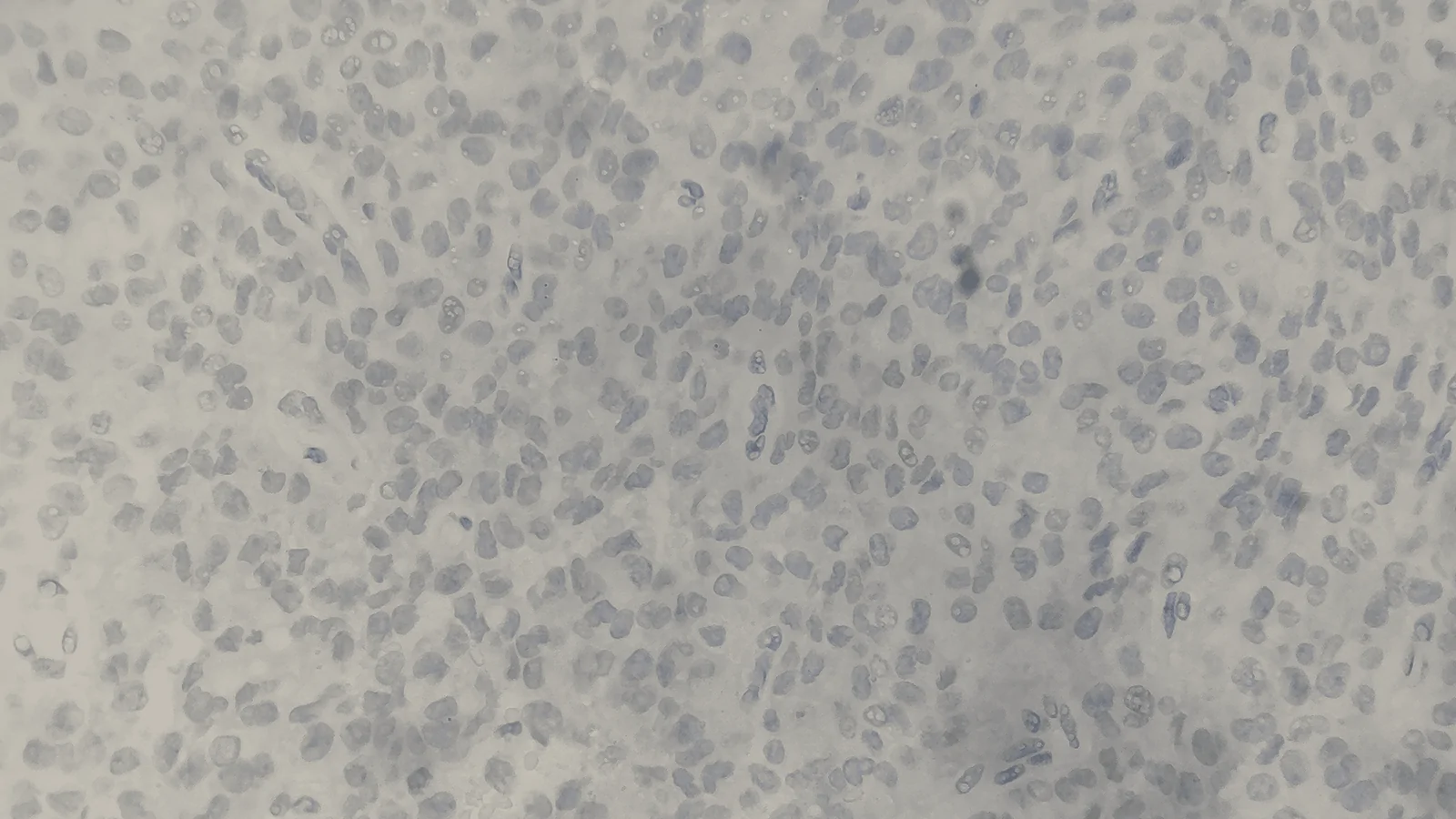
Histopatholgy imaging showing loss of E-cadherin in the tumour cells

A transient biochemical pancreatic fistula developed postoperatively but resolved spontaneously, and the patient remains disease-free after one year of follow-up.

Case two

A 33-year-old woman with a known history of epilepsy and on antiepileptics presented with a 15-day history of intermittent epigastric pain and four days of recurrent vomiting, without jaundice, fever, or weight loss. Physical examination was unremarkable, and all haematological and biochemical parameters, including serum amylase, lipase, and CA 19.9, were within normal limits (Table 1).

Contrast-enhanced CT demonstrated a 7.4 × 6.4 × 5.3 cm well-circumscribed lobulated solid cystic mass in the pancreatic body containing calcific foci and compressing the splenic vein and splenoportal confluence, suggestive of SPEN (Figure 5).

**Figure 5:**
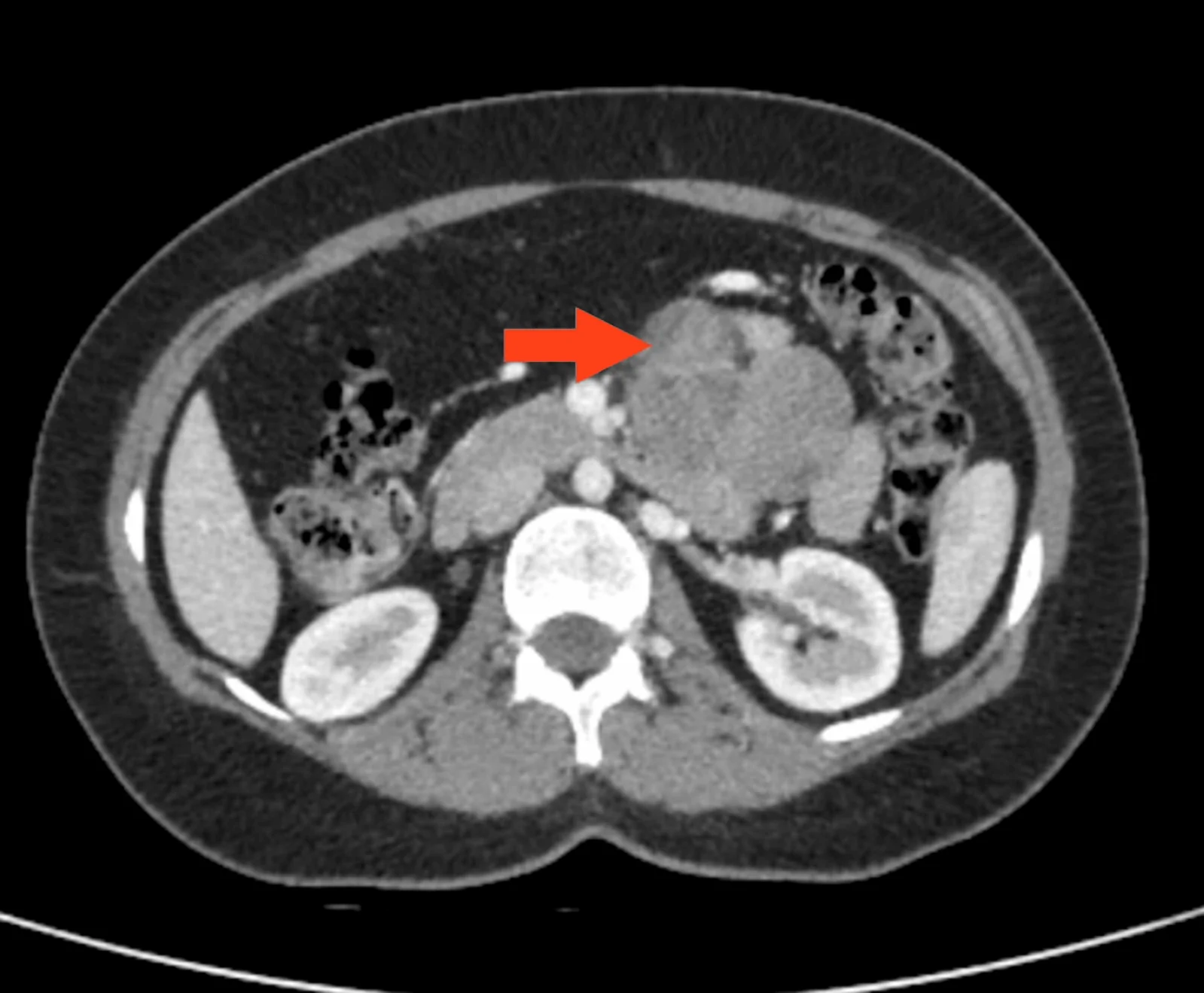
Contrast-enhanced CT demonstrated well-circumscribed lobulated solid-cystic mass in the pancreatic body containing calcific foci and compressing the splenic vein (red arrow indicates the tumour)

Following informed consent, exploratory laparotomy was performed, and due to the tumour’s intimate relation to major vascular structures, distal pancreatectomy with splenectomy was undertaken. Gross examination revealed a well-defined solid cystic lesion measuring 8.5 × 5.5 × 2.6 cm within a 10.5 × 7.2 × 3.2 cm partial pancreatectomy specimen (Figure 6).

**Figure 6:**
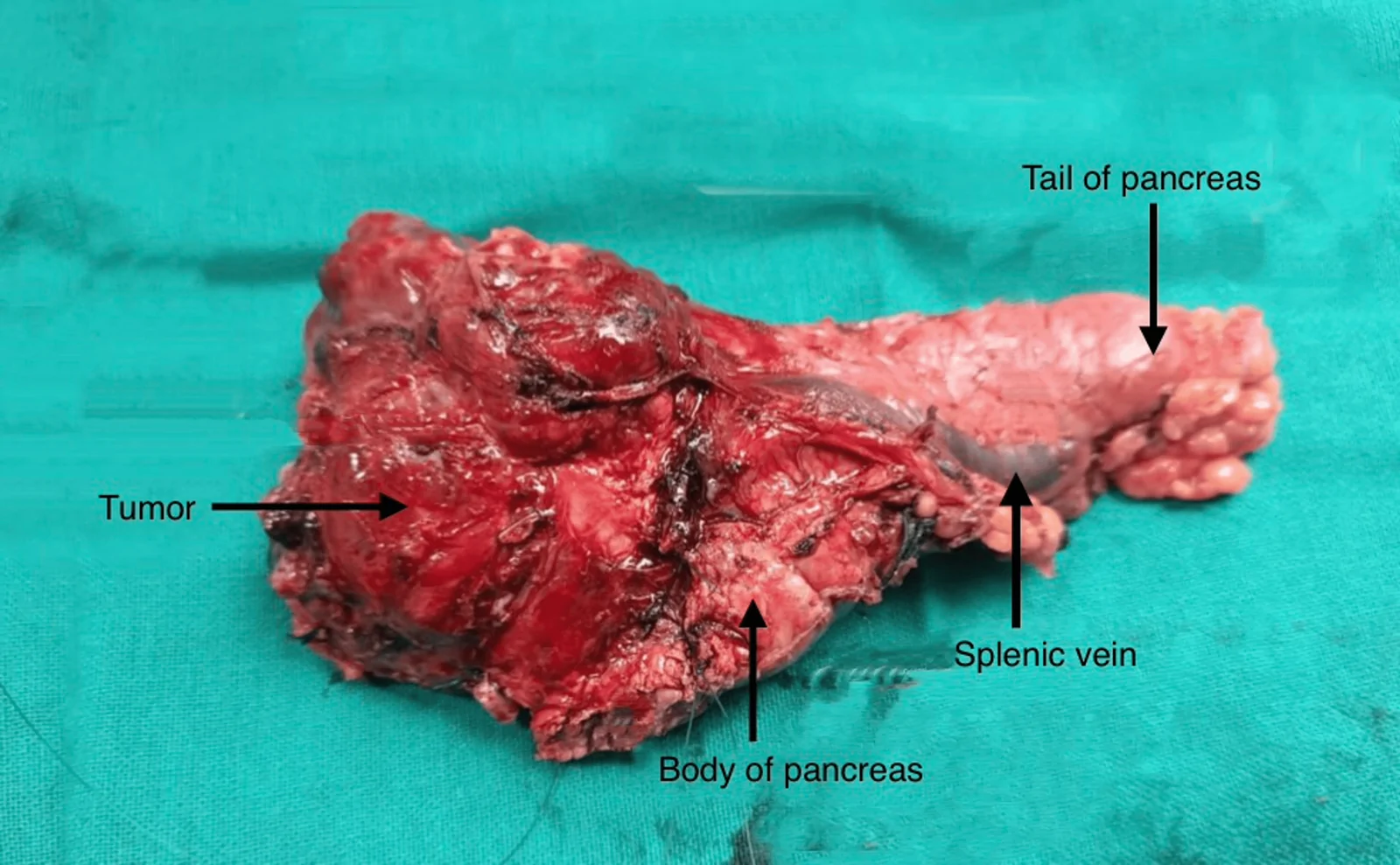
Gross specimen showing the large pancreatic lesion with the splenic vein

Microscopic evaluation showed characteristic solid and pseudopapillary patterns with uniform eosinophilic cells, necrotic and haemorrhagic foci, while immunohistochemistry demonstrated nuclear β-catenin and progesterone receptor positivity with loss of E-cadherin, confirming SPEN. The tumour was staged as pT3 N0 Mx (American Joint Committee on Cancer (AJCC) 8th edition [7]). The postoperative course was uneventful, and the patient remains disease-free on follow-up.

## Discussion

SPEN is an uncommon, low-grade malignant tumour constituting approximately 1-2% [2] of all exocrine pancreatic neoplasms. It exhibits a striking predilection for young women, while cases occurring in males or older individuals tend to display a more aggressive clinical course. Although the exact cellular origin remains uncertain, consistent mutations in the CTNNB1 gene leading to dysregulation of the Wnt/β-catenin signalling pathway are recognised as pivotal molecular events in tumorigenesis [5,6]. These mutations promote nuclear accumulation of β-catenin and loss of membranous E-cadherin, disrupting intercellular adhesion and imparting the characteristic pseudopapillary architecture [6]. The frequent expression of progesterone receptors and pronounced female predominance further support the hypothesis of hormonal influence in SPEN pathobiology.

Radiologically, SPENs typically present as large, well-encapsulated, and sharply demarcated masses with mixed solid and cystic components, resulting from intra-tumoural haemorrhage and degenerative changes [8]. On CT or MRI, these lesions often show peripheral enhancement, internal septations, or coarse calcifications, helping differentiate them from pancreatic neuroendocrine or mucinous cystic neoplasms [9,10]. When imaging findings are inconclusive, endoscopic ultrasound-guided fine-needle aspiration (EUS-FNA) provides diagnostic accuracy of up to 85% [3]. Histopathologically, SPENs exhibit solid and pseudopapillary structures with delicate fibrovascular cores lined by monomorphic epithelial cells possessing eosinophilic cytoplasm and nuclear grooves [11]. Immunohistochemically, they display nuclear β-catenin, vimentin, CD10, and progesterone receptor positivity, while lacking neuroendocrine markers such as chromogranin and synaptophysin, features that were confirmed in both patients of this series.

Surgical excision remains the definitive and curative treatment for SPEN, with the choice of procedure dictated by tumour location and its relationship to major vascular and ductal structures [3]. Central pancreatectomy is preferred for mid-body lesions to preserve pancreatic parenchyma and function, while distal pancreatectomy, with or without splenectomy, is recommended for tumours of the pancreatic body and tail. Enucleation may be considered for small, well-encapsulated lesions not involving the main pancreatic duct, and minimally invasive techniques, laparoscopic or robotic, have demonstrated comparable oncologic outcomes with reduced morbidity. The overall prognosis following complete (R0) resection is excellent, with five-year survival exceeding 95% [3]. Recurrence occurs in only 10-15% of patients, commonly within five years, and is associated with larger tumours, capsular invasion, or positive margins [11,12]. Even in metastatic or locally invasive disease, surgical intervention may yield prolonged survival. Both patients discussed here underwent successful resections, one underwent laparoscopic central pancreatectomy with splenic preservation and the other distal pancreatectomy with splenectomy, and remain disease-free, underscoring the favourable long-term outcomes achievable with early detection and complete excision.

## Conclusions

In conclusion, SPEN represents a rare but distinct pancreatic neoplasm with an excellent prognosis when identified early and treated surgically. Accurate diagnosis, supported by radiological and immunohistochemical correlation, is essential for appropriate management. Complete resection with vigilant follow-up ensures durable cure and near-universal long-term survival. In our two cases, both patients were young women with pancreatic body tumors and characteristic solid-cystic features on imaging. Surgical management was individualized according to tumor location and vascular involvement, with both patients achieving complete resection and favorable postoperative outcomes. These cases highlight the importance of early recognition, accurate diagnosis, and appropriate surgical planning in the successful management of SPEN.
